# Bovine Immunoglobulin/Protein Isolate Binds Pro-Inflammatory Bacterial Compounds and Prevents Immune Activation in an Intestinal Co-Culture Model

**DOI:** 10.1371/journal.pone.0120278

**Published:** 2015-04-01

**Authors:** Christopher J. Detzel, Alan Horgan, Abigail L. Henderson, Bryon W. Petschow, Christopher D. Warner, Kenneth J. Maas, Eric M. Weaver

**Affiliations:** 1 Entera Health, Inc., Ankeny, Iowa, United States of America; 2 Entera Health, Inc., Cary, North Carolina, United States of America; Massachusetts General Hospital, UNITED STATES OF AMERICA

## Abstract

Intestinal barrier dysfunction is associated with chronic gastrointestinal tract inflammation and diseases such as IBD and IBS. Serum-derived bovine immunoglobulin/protein isolate (SBI) is a specially formulated protein preparation (>90%) for oral administration. The composition of SBI is greater than 60% immunoglobulin including contributions from IgG, IgA, and IgM. Immunoglobulin within the lumen of the gut has been recognized to have anti-inflammatory properties and is involved in maintaining gut homeostasis. The binding of common intestinal antigens (LPS and Lipid A) and the ligand Pam3CSK4, by IgG, IgA, and IgM in SBI was shown using a modified ELISA technique. Each of these antigens stimulated IL-8 and TNF-α cytokine production by THP-1 monocytes. Immune exclusion occurred as SBI (≤50 mg/mL) bound free antigen in a dose dependent manner that inhibited cytokine production by THP-1 monocytes in response to 10 ng/mL LPS or 200 ng/mL Lipid A. Conversely, Pam3CSK4 stimulation of THP-1 monocytes was unaffected by SBI/antigen binding. A co-culture model of the intestinal epithelium consisted of a C2BBe1 monolayer separating an apical compartment from a basal compartment containing THP-1 monocytes. The C2BBe1 monolayer was permeabilized with dimethyl palmitoyl ammonio propanesulfonate (PPS) to simulate a damaged epithelial barrier. Results indicate that Pam3CSK4 was able to translocate across the PPS-damaged C2BBe1 monolayer. However, binding of Pam3CSK4 by immunoglobulins in SBI prevented Pam3CSK4 translocation across the damaged C2BBe1 barrier. These results demonstrated steric exclusion of antigen by SBI which prevented apical to basal translocation of antigen due to changes in the physical properties of Pam3CSK4, most likely as a result of immunoglobulin binding. This study demonstrates that immunoglobulins in SBI can reduce antigen-associated inflammation through immune and steric exclusion mechanisms and furthers the mechanistic understanding of how SBI might improve immune status and reduce inflammation in various intestinal disease states.

## Introduction

The intestinal epithelial barrier is critical in maintaining homeostasis of the gastrointestinal (GI) tract. Dysfunction of the intestinal epithelium is characterized by loss of barrier function, antigen translocation, inflammation of intestinal tissue, and nutrient malabsorption. Such alterations contribute to enteropathy associated with diseases such as diarrhea-predominant irritable bowel syndrome (IBS-D), inflammatory bowel disease (IBD), and HIV-associated-enteropathy [1–3]. The persistent nature of the enteropathy associated with these conditions can be attributed to the cyclical cause and effect relationship of an altered gut microbiota, gut barrier dysfunction, and immune activation with each contributing factor cascading into the next, resulting in a progressive loss of gut homeostasis and chronic enteropathy.

Enteropathy associated with these GI diseases is most likely not attributable to a single factor, but is rather the result of the combined effects of genetic susceptibility, exposure to environmental pathogens or toxins, abnormal bile acid metabolism, diet or other physiological factors. Any combination of such factors can contribute to dysregulated immunity and aberrant cytokine production, which have been shown to compromise the structural integrity of the intestinal epithelium [4]. Impairment of the epithelial barrier allows luminal antigens access to the immunoreactive cells within the lamina propria, resulting in inflammation [5]. Accordingly, it can be assumed that intestinal inflammation can be attenuated by retaining antigens in the intestinal lumen as well as limiting the ability of antigens to interact with immune cells, ultimately reducing inflammatory cytokine production which serves to limit enteropathy and support gut homeostasis.

It has long been accepted that oral immunoglobulins play a critical role in gut homeostasis. Immunoglobulins in colostrum and breast milk confer passive immunity, have anti-inflammatory properties, and contribute to the establishment of the intestinal microbiota and gut barrier integrity [6–9]. Furthermore, studies have shown that bovine immunoglobulin binds and neutralizes a wide array of microbial components [10,11], has the capacity to positively affects the gut microbiota [12], and help maintain proper intestinal barrier function [13,14]. Petschow et al. provides a comprehensive review of pre-clinical and clinical studies detailing the proposed mechanism of action for the role of oral immunoglobulins in maintaining gastrointestinal homeostasis [15]. In addition, a recent paper highlights the concept that administration of oral immunoglobulin provides for a “distinctive nutritional requirement” needed in management of various enteropathies [16].

Serum-derived bovine immunoglobulin/protein isolate (SBI) is a specially-formulated protein preparation designed for oral administration to aid in the management of chronic loose and frequent stools. SBI is an enriched bovine plasma fraction in which the immunoglobulin content has been increased from near 13% of the protein in liquid plasma to a formulation where IgG is greater than 50% of the total proteins [15]. Likewise, IgA and IgM immunoglobulins have also been enriched in SBI to 1% and 5% of the total proteins, respectively, bringing the total immunoglobulin content of SBI to ~60%. SBI has previously been shown to improve GI homeostasis and the symptoms associated with HIV enteropathy as measured by reduced number of bowel movements and improved stool consistency [12]. Wilson et al. has also shown that SBI improves GI symptoms (e.g. abdominal pain, flatulence, bloating, urgency, loose stools) in subjects with IBS-D [17]. The improvements in GI function and homeostasis attributed to oral immunoglobulin have been shown to be a result of reduced epithelial macromolecular flux, improved intestinal barrier function, and reduced colonic permeability [13,14]. However, the exact role of oral immunoglobulins in the physiological improvement of GI function has yet to be completely elucidated.

To expand our understanding of the mechanisms responsible for the benefits of oral immunoglobulin on alleviating GI symptoms, we studied the epithelial translocation of antigens using a co-culture model of the intestinal barrier. The co-culture model consisted of a C2BBe1 epithelial cell barrier that separated an apical compartment from a basal compartment containing THP-1 monocytes. The production of inflammatory cytokines by the THP-1 cells in response to antigen exposure was measured to better understand how immunoglobulins modulate the translocation of antigens across an epithelial membrane. Our results demonstrate the binding affinity of the different immunoglobulin isotypes (IgG, IgA, and IgM) in SBI for LPS, Lipid A, and Pam3CSK4. These antigens were chosen based on their ubiquitous presence in the lumen of the gut and varied size and charge characteristics. We also demonstrate the role of the immunoglobulins in SBI in immune exclusion and steric exclusion of antigen-induced inflammatory cytokine production.

## Materials and Methods

### Antigen Binding ELISA

Bovine immunoglobulin antibodies specific for *E*. *coli* K12 LPS, Lipid A and PAM3CSK4 (Invivogen, San Diego, CA) were detected using a modified ELISA technique. Navarro et al. previously employed this same modified ELISA technique to assess bovine immunoglobulin binding to LPS core polysaccharides [10]. High binding ELISA plates (Thermo Fisher Scientific, Waltham, MA) were coated overnight with 1 μg of antigen by adding 100μl of 10 μg/mL antigen in ELISA Coating Buffer (Biolegend, San Diego, CA). Wells were then washed 3X with Dulbecco’s phosphate-buffered saline (DPBS, Life Technologies, Grand Island, NY) containing 0.5% Tween-20 (PBS-Tween). Each well was then blocked by adding 300 μl of 1% (wt/vol) BSA (Proliant Biologicals, Boone, IA) in PBS-Tween for 30 min at 37°C, followed by three additional washes with PBS-Tween. SBI or control protein samples were then added to the appropriate wells (100μl/well) and incubated for 1 h at 37°C. Samples were then removed and discarded and wells washed 3X with PBS-Tween. Antigen binding by SBI immunoglobulin isotypes was detected by adding 100μl/well of sheep anti-bovine IgG, anti-bovine IgA, or anti-bovine IgM labeled with HRP (Bethyl, Montgomery, TX) at a dilution of 1:100,000 followed by incubation for 1 h at 37°C. Wells were again washed 3X with PBS-Tween and then incubated with 100μl of TMB substrate (Bethyl, Montgomery, TX) at room temperature for 10 minutes. The colorimetric reaction was stopped by the addition of 50μl of 0.2M H_2_S0_4_. The absorbance of each well was measured at 450nm using a microplate reader (Molecular Devices, Sunnyvale, CA).

Control ELISA experiments were conducted to determine the non-specific binding of the HRP-conjugated detection antibodies and the specific recognition of antigen by the immunoglobulins in SBI. Non-specific binding by HRP-conjugated detection antibodies was tested by excluding SBI from the ELISA protocol followed by absorbance measurement. Furthermore, the efficiency of the blocking protein was tested to show specificity of the immunoglobulins in SBI for the test antigens by measuring absorbance in unblocked wells and wells not coated with antigen. Functional binding of the immunoglobulins in SBI with antigen was investigated using denatured SBI. SBI was denatured by boiling for 15 minutes and then immediately cooled on ice to ensure all proteins remained unfolded prior to use in the modified ELISA protocol.

### Cell Culture

Cell culture reagents including high glucose Dulbecco’s Modified Eagle Medium (DMEM), Roswell Park Memorial Institute 1640 (RPMI 1640), and penicillin-streptomycin were purchased from Life Technologies (Grand Island, NY). Fetal bovine serum (FBS) was purchased from Sigma-Aldrich (St. Louis, MO). LPS from *E*. *coli* K12, Lipid A, and Pam3CSK4 antigens were obtained from Invivogen (San Diego, CA).

C2BBe1 cells, a clone of the human intestinal Caco-2 cell line, was purchased from ATCC (ATCC CRL-2102, Manassas, VA) and maintained in tissue culture treated flasks (Corning Costar, Cambridge, MA, USA) with DMEM containing 10% FBS, 50 U/ml penicillin, 50 U/ml streptomycin. Cells were maintained at 37°C in a 5% CO_2_ humidified atmosphere and sub-cultured after becoming 70–80% confluent. Co-culture studies were conducted using the 24-well HTS-Transwell Culture System (Corning Costar, Cambridge, MA). C2BBe1 cells were seeded onto the semi-permeable membranes of the HTS plates at a density of 3.2 x 10^4^ cells/well, and maintained for 21-days with culture medium exchanged every 3–4 days. On day 21, transepithelial electrical resistance (TER) of the C2BBe1 monolayer was measured using the Millicell-ERS electrical resistance measuring system (Millipore, Bedford, MA) and chopstick electrodes (Millipore). TER measurements were conducted with 1.25mL of DMEM in the basal compartment and 0.25 mL of DMEM in the apical compartment. C2BBe1 monolayers having a TER greater than 250 Ω·cm^2^ were used in co-culture studies of steric exclusion.

The THP-1 human monocyte cell line was purchased from ATCC (ATCC TIB-202, Manassas, VA) and maintained in RPMI 1640 supplemented with 10% fetal bovine serum, 50 U/ml penicillin, and 50 U/ml streptomycin at 37°C in tissue culture treated flasks, and sub-cultured as needed.

### Preparation of Protein Solutions

SBI and collagen protein solutions used in the testing of immune and steric exclusion mechanisms were prepared by dissolving 50 mg/mL SBI (Entera Health, Cary, NC), or collagen (Proliant Health, Ankeny, IA), in DMEM. Protein solutions were sterile filtered through a 0.22 μm PES syringe filter (EMD Millipore, Billerica, MA) prior to use in cell culture.

### Immune Exclusion

Immune exclusion experiments were conducted to evaluate the impact of immunoglobulins in SBI on the production of antigen-induced inflammatory cytokines by THP-1 monocytes. THP-1 cells were seeded into 24-well tissue culture plates (1 mL of 3x10^5^ THP-1 cells/mL was added to each well) and incubated for 24 hours prior to experimentation. THP-1 cytokine production in response to various antigens was determined by adding antigen solution to each well, followed by measurement of media cytokine concentrations 24 hours after antigen addition. The immune exclusion properties of SBI and a control protein (collagen) were assessed by adding LPS (10 ng/mL), Lipid A (200 ng/mL), or Pam3CSK4 (10 ng/mL) to DMEM solutions containing protein (SBI or collagen) at concentrations up to 50 mg/mL followed by incubation for 1 hour at 37°C prior to addition to cell culture. THP-1 cells were incubated with antigen/protein mixtures for 24 hours followed by cytokine analysis of the culture medium to assess the immune exclusion properties of SBI and collagen.

### Steric Exclusion

C2BBe1 monolayers (TER >250 Ω*cm^2^) were prepared for steric exclusion experiments by transferring the HTS transwell inserts to a 24-well plate containing 3x10^5^ THP-1 cells/well, with the apical compartment containing 0.25 mL of media and the basal compartment containing THP-1 cells in 1.25 mL of media. A similar model was developed by Watanabe et al. to study Caco-2 monolayer damage induced by differentiated THP-1 macrophages [18].

Apical to basal antigen translocation was functionally assessed by measuring IL-8 production by THP-1 cells maintained in the basal compartment. Steric exclusion experiments were conducted by adding antigen (LPS, 10 ng/mL; Pam3CSK3, 10 ng/mL; Lipid A, 200 ng/mL) or antigen combined with SBI or control protein at varying concentrations up to 50 mg/mL to the apical compartment of the co-culture system. Antigen/protein combinations were incubated for 1 hour at 37°C prior to addition to the apical compartment, in order to allow protein antigen binding. The translocation of antigen and antigen/protein complexes across intact and 0.01% dimethyl palmitoyl ammonio propanesulfonate (PPS, Sigma, St. Louis, MO) damaged C2BBe1 monolayers was assessed by measuring basal THP-1 monocyte cytokine production following a 24 hour incubation. PPS has previously been studied as a membrane permeability enhancer and has been shown affect tight junction permeability [19].

### Cytokine Analysis

Cell-free media supernatants were assayed for pro-inflammatory cytokines, IL-6, IL-8, and TNF-α using a Bio-Plex MAGPIX Multiplex Reader and Bio-Plex Pro Assays (Bio-Rad, Hercules, CA). Bio-Plex Pro Assays to measure cytokine concentrations in cell culture medium were conducted according to the manufacturer’s instructions.

### Statistical Analysis

All data are reported as the mean ± the standard deviation. Statistical comparisons were performed using Prism version 6.04 (Graph Pad Software Inc, San Diego, CA). Statistical significant differences between treatment groups were determined using the Student’s t-test and defined as P<0.05.

## Results

### Antigen Binding

**Fig 1:**
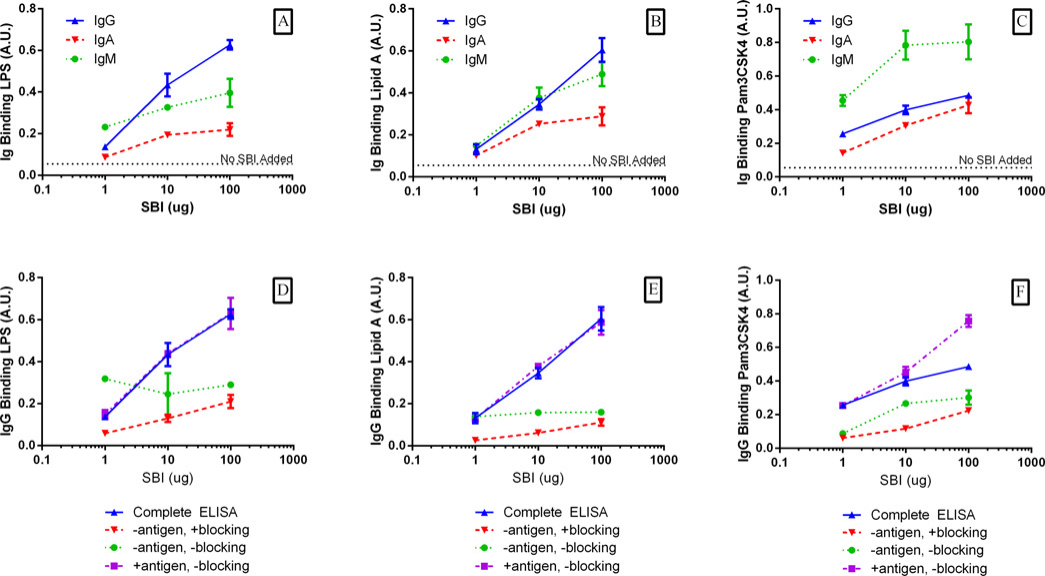
Binding Affinities of Immunoglobulins in SBI for Test Antigens. The binding affinities of bovine IgG, IgM, and IgA for LPS (Panel A), Lipid A (Panel B), and Pam3CSK4 (Panel C) were demonstrated using a modified ELISA technique. ELISA control conditions (D-F) confirmed IgG had a greater affinity for the antigens of interest (Complete ELISA) when compared with non-specific binding to blocking proteins (-antigen, +blocking) or the surface of the ELISA plate (-antigen,-blocking).

The binding of antigen by immunoglobulins in SBI was qualitatively demonstrated using a modified ELISA technique [10]. The binding of IgG, IgA, and IgM in SBI for LPS from *E*. *coli* K12, Lipid A, and Pam3CSK4 are shown in Fig. 1A-C. The final measured absorbance was directly proportional to SBI concentration, indicating that increased SBI concentrations correlated with greater binding of immunoglobulin to immobilized antigen as shown by higher absorbance readings. Since the affinities of the anti-IgG, anti-IgA, and anti-IgM detection antibodies for the various immunoglobulin isotypes were unknown, a relative comparison of isotype affinity for a particular antigen could not be inferred.

Specificity of immunoglobulin binding to antigen was verified by testing several control conditions. Control tests were conducted by following the modified ELISA protocol but omitting specific steps in order to evaluate non-specific immunoglobulin binding at various steps throughout the testing protocol. The IgG isotype binding controls for LPS (Fig. 1D), Lipid A (Fig. 1E), and Pam3CSK4 (Fig. 1F) showed higher affinity of IgG for antigen (+antigen,-blocking) compared to nonspecific binding within the ELISA well (-antigen,-blocking) or with the blocking protein (-antigen, +blocking). Similar results were obtained with binding controls for IgA (S1 Fig.) and IgM (S2 Fig.).

Additional experiments were conducted to test the non-specific binding of HRP-conjugated detection antibodies with test antigens and the specific recognition of antigen by the immunoglobulins in SBI. Omitting SBI from the ELISA protocol resulted in complete elimination of antigen binding signal, indicating detection antibodies did not bind with test antigens (S3–S5 Figs.). The specificity of the immunoglobulin/antigen interaction was also confirmed using denatured SBI in experiments. The addition of denatured SBI resulted in the absence of signal in the modified ELISA, indicating that the immunoglobulins in SBI must remain in their native conformation for antigen binding to occur. These control tests of the modified ELISA specificity are shown for IgG (S3 Fig.), IgA (S4 Fig.), and IgM (S5 Fig.) as Supporting Information. In summary, results from control ELISA experiments combined with results from experiments with individual antigens (LPS, Lipid A, and Pam3CSK4) in the complete ELISA model demonstrate specific antigen recognition and binding by each immunoglobulin isotype contained in SBI (IgG, IgA, and IgM).

### Immune Exclusion

**Fig 2:**
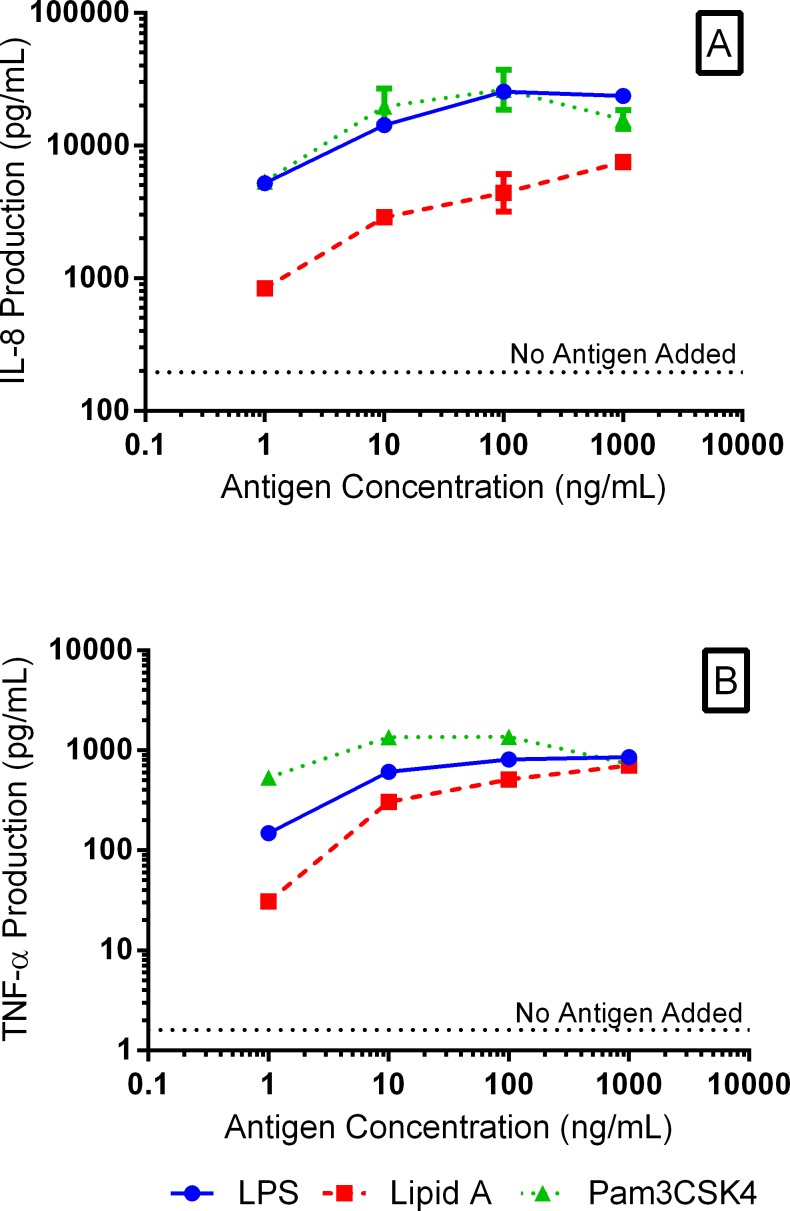
THP-1 Cytokine Response to Test Antigens. THP-1 monocytes were extremely sensitive to activation by LPS, Lipid A, and Pam3CSK4. Production of IL-8 (A) and TNF-a (B) was observed at antigen concentrations as low as 1 ng/mL and cytokine production increased with increasing antigen concentration in a dose-dependent manner.

Testing for the potential immune exclusion mechanism required the demonstration of SBI binding to antigen and subsequent inhibition of inflammatory cytokine production by immune cells. THP-1 monocytes produce a wide range of inflammatory cytokines and differentiate toward a macrophage lineage in response to antigenic stimuli [20]. The production of IL-6, TNF-α, and IL-8 cytokines by THP-1 cells in response to each antigen was measured and results are shown in Fig. 2. Production of IL-6 was not detected in response to any of the antigens at the concentrations tested in this study. However, production of IL-8 (Fig. 2A) and TNF-α (Fig. 2B) by THP-1 monocytes was shown to increase in a dose dependent manner in response to increasing concentrations of LPS and Lipid A ranging from 1 ng/mL to 1000 ng/mL. A similar trend in the production of IL-8 and TNF-α was observed as Pam3CSK4 concentration increased from 1 ng/mL to 100 ng/mL, while production of both IL-8 and TNF-α tended to decrease when Pam3CSK4 was tested at 1000 ng/mL (Fig. 2). Decreasing cytokine production at high Pam3CSK4 concentrations may indicate negative feedback regulation of inflammatory cytokine production at high antigen concentrations, a hypothesis needing confirmation through further experimentation.

Lower concentrations of LPS and Pam3CSK4 (10 ng/mL) were used in immune and steric exclusion experiments, when compared to Lipid A (200 ng/mL), presumably due to the decreased sensitivity of THP-1 monocytes to Lipid A stimulation. These concentrations are below peak cytokine production levels in order to maintain cell sensitivity to changing antigen concentrations and avoid any misinterpretation in antigen binding due to a plateau in inflammatory cytokine production near maximum antigen concentrations. This ensures that small changes in antigen availability due to immunoglobulin binding are detectable.

For each of the antigens tested, IL-8 and TNF-α followed the same trend with IL-8 being consistently expressed at the higher level. Similar expression profiles of IL-8 and TNF-α were expected as these are early cytokine markers of immune activation and both cytokines are under the control of NF-κB activation, a primary pathway in the production of inflammatory cytokines in response to antigen stimulation [21]. Therefore, IL-8 was solely monitored in subsequent experiments to determine antigen activation of THP-1 monocytes.

To test the immune exclusion properties of the immunoglobulins in SBI, antigens were initially incubated with SBI or control protein (collagen) for a minimum of 1 hour to allow immunoglobulin/antigen binding. Binding within this time frame was previously shown for each immunoglobulin isotype and antigen combination in the modified ELISA results (Fig. 1). Since it is inherently difficult to extrapolate the *in vivo* physiologic relevance of any *in vitro* testing system, levels of SBI and control protein that were chosen for testing in these studies (0.8 mg/mL-50 mg/mL) were based on concentrations that elicited a linear response in antigen binding in preliminary experiments.

**Fig 3:**
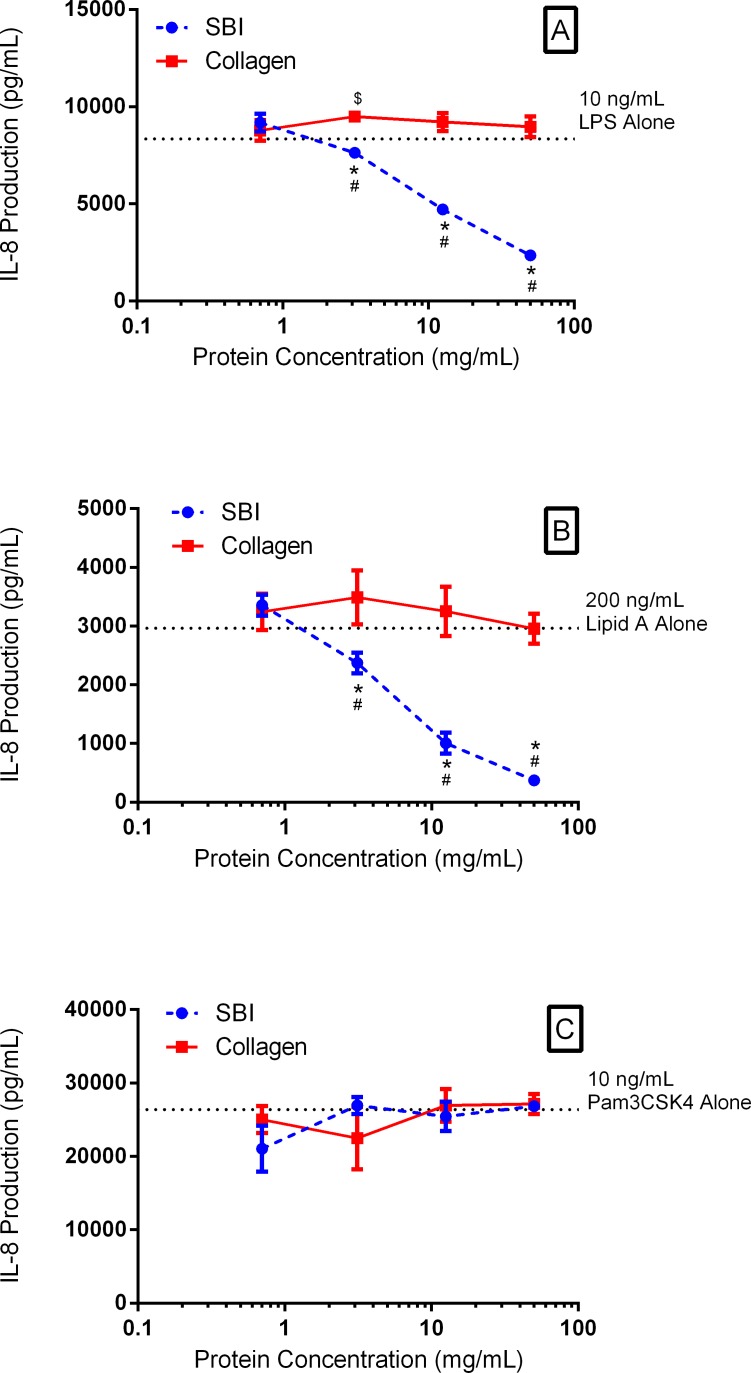
Immune Exclusion of Test Antigens. Immune exclusion of LPS (A) and Lipid A (B) induced IL-8 pro-inflammatory cytokine production was observed by the addition of SBI. Cytokine production by LPS and Lipid A was increasingly inhibited as the SBI concentration was increased from 1 mg/mL to 50 mg/mL. Immune exclusion of Pam3CSK4 (C) was not observed as the addition of SBI did not affect cytokine production. The control protein collagen did not affect cytokine production induced by any test antigen. * indicates P<0.05, when comparing the difference between SBI and collagen. # indicates P<0.05, when comparing the immune exclusion of SBI at varying concentrations to the amount of IL-8 produced when the antigen is added alone. $ indicates P<0.05, when comparing the immune exclusion of collagen at varying concentrations to the amount of IL-8 produced when the antigen is added alone.

Immune exclusion of LPS and Lipid A by immunoglobulins in SBI was demonstrated by the decreased production of IL-8 as the concentration of added SBI was increased from 0.8 mg/mL to 50 mg/mL (Fig. 3A-B). At the lowest test concentration of SBI, 0.8 mg/mL, the production of IL-8 in response to 10 ng/mL LPS was unchanged (Fig. 3A). However, increasing SBI concentration to 3.125 mg/mL resulted in a 17% reduction in LPS-induced IL-8 production while further increases in SBI concentration (as high as 50 mg/mL) resulted in a greater than 70% inhibition of IL-8 production (Fig. 3A). In similar fashion, SBI inhibited IL-8 production by THP-1 monocytes that were exposed to Lipid A at 200 ng/mL (Fig. 3B). For both LPS and Lipid A, the minimum SBI concentration required to significantly reduce IL-8 production was 3.125 mg/mL. Conversely, control protein (collagen) was unable to significantly reduce IL-8 production in response to either LPS or Lipid A, as shown by constant production of IL-8 by THP-1 monocytes at collagen concentrations as high as 50 mg/mL (Fig. 3). Interestingly, SBI and control protein collagen were both unable to significantly inhibit Pam3CSK4-mediated IL-8 production at protein concentrations as high as 50 mg/mL (Fig. 3C). The absence of any immune exclusion properties of Pam3CSK4 by the immunoglobulins in SBI was surprising given that IgG, IgA, and IgM were all shown to bind Pam3CSK4 using the modified ELISA method (Fig. 1C).

### Co-Culture Permeability

To approximate a damaged epithelial barrier, the permeability of the C2BBe1 monolayer was increased with the addition of PPS, which was previously described to control intestinal therapeutic absorption [19]. The addition of PPS to the apical side of C2BBe1 monolayers decreased TER in a concentration- and time-dependent manner, similar to trends observed by Gupta et al. when PPS was added to Caco-2 cell monolayers [19]. A PPS concentration of 0.01% in media was chosen to permeabilize cell monolayers since this concentration irreversibly reduced the TER of mature monolayers from 250–350 Ω*cm^2^ to near 100 Ω *cm^2^, representing a 60–70% reduction of the initial TER value.

**Fig 4:**
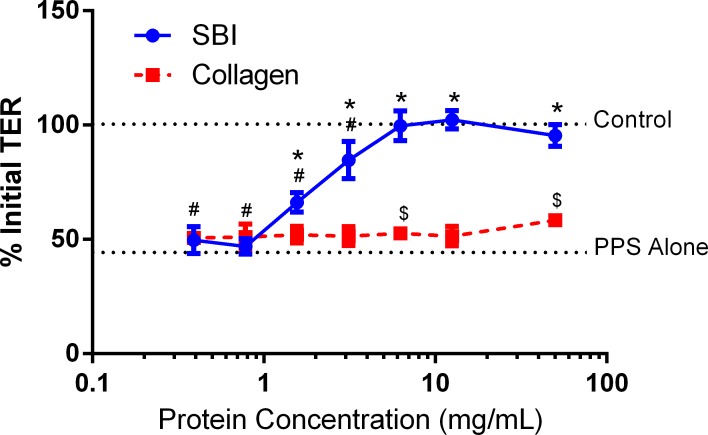
Damaging the Co-Culture C2BBe1 Monolayer. Addition of 0.01% PPS to the apical compartment C2BBe1 transwell culture plates resulted in a 60–70% reduction in TER. The addition of SBI inhibited the PPS-induced loss in TER as SBI concentrations were increased from 0.8 mg/mL, with complete inhibition of PPS-induced barrier damage observed at concentrations above 6.25 mg/mL. Inhibition of PPS-induced loss of TER required the functional proteins in SBI, as the control protein collagen had no effect on PPS induced barrier damage. * indicates P<0.05, when comparing the difference between SBI at varying concentrations and PPS alone. # indicates P<0.05, when comparing the difference between SBI at varying concentrations and the control TER value. $ indicates P<0.05, when comparing the difference between collagen at varying concentrations and PPS alone.

While the concentration and time dependent effects of PPS on monolayer permeability were expected, it was unexpected that SBI was able to mitigate PPS-induced membrane damage (Fig. 4). Concentrations of SBI greater than 1.5 mg/mL significantly inhibited PPS-induced reduction in TER, with 0% reduction in TER being observed at SBI concentration of 6.25 mg/mL and greater (Fig. 4). The control protein collagen was incapable of inhibiting PPS-induced C2BBe1 cell monolayer damage (Fig. 4). While PPS is known to affect the tight junctions of epithelial membranes to increase barrier permeability, the exact mechanism is currently unknown [19]. Results from these experiments suggest that the functional proteins in SBI are required for the observed inhibition of PPS-induced membrane damage. Future work will determine the mechanism of this effect.

**Fig 5:**
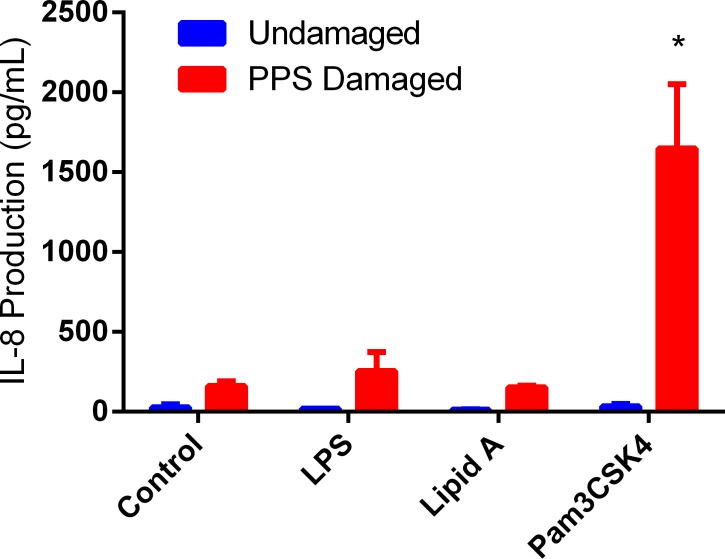
Translocation of Test Antigens Across Damaged Co-Culture Monolayers. Each test antigen was added to the apical compartment of undamaged and PPS damaged C2BBe1 co-cultures. LPS, Lipid A, and Pam3CSK4 were added at 10 ng/mL, 200 ng/mL, and 10 ng/mL, respectively. Pam3CSK4 was the only antigen tested capable of translocating a PPS-damaged barrier and stimulating IL-8 production by basal THP-1 monocytes. The small increases in IL-8 production observed for both LPS and Lipid A added to the apical compartment were not statistically different from PPS alone indicating these antigens were unable to translocate the PPS damaged barrier. * indicates P<0.05, when comparing PPS-treated controls with varying antigen treatments.

The permeability of C2BBe1 monolayers was tested following the addition of antigen to the apical compartment of undamaged and damaged membranes. C2BBe1 monolayers having a TER value between 250–350 Ω *cm^2^ were impermeable to antigen translocation, as shown by the absence of IL-8 production by basal THP-1 monocytes following the addition of LPS, Lipid A, or Pam3CSK4 to the apical compartment of intact monolayers (Fig. 5). Permeabilization of the C2BBe1 monolayers with 0.01% PPS resulted in a moderate increase in the production of IL-8 by basal THP-1 cells from background levels of 28 ± 18 pg/mL to 161 ± 30 pg/mL (Fig. 5, Control, PPS Damaged). No significant difference (P<0.05) in IL-8 production was observed between PPS-damaged control, LPS (255 ± 119 pg/mL), or Lipid A (153 ± 9) cell cultures (Fig. 5), indicating that neither LPS nor Lipid A was able to translocate the PPS-damaged monolayers. However, Pam3CSK4 was able to translocate across damaged membrane as shown by a significant increase in IL-8 production following Pam3CSK4 addition to the apical compartment of PPS-damaged monolayer (Fig. 5).

### Steric Exclusion

Steric exclusion refers to a mechanism where immunoglobulin binding of antigen alters the physical characteristics of the antigen and inhibits translocation of the antigen across an epithelial barrier. Physical properties which might be altered through immunoglobulin binding include qualities such as size, charge, and hydrophobicity; each of which can contribute to an antigen’s translocation efficiency. Experiments to evaluate steric exclusion of antigens by SBI was limited to studying Pam3CSK4 since this was the only antigen tested that was capable of translocating across PPS-damaged C2BBe1 membranes.

**Fig 6:**
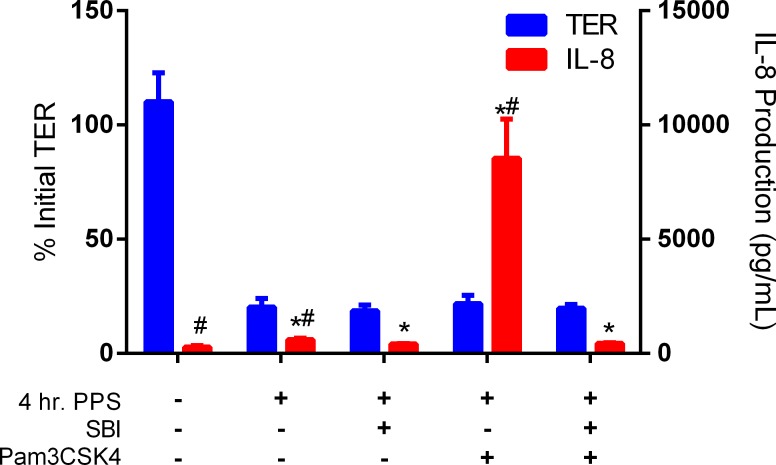
Steric Exclusion of Pam3CSK4 by Immunoglobulin Binding. Pam3CSK4 translocation across a PPS damaged epithelial barrier was inhibited by steric exclusion when 10 ng/mL of Pam3CSK4 was bound by the immunoglobulin in 50 mg/mL of SBI. All co-cultures were incubated with 0.01% PPS for 4 hours prior to experimentation to ensure epithelial barriers were damaged. Pam3CSK4 translocates from the apical to basal compartment across a PPS damaged epithelial barrier. Binding of Pam3CSK4 by the immunoglobulins in SBI completely inhibits antigen translocation as shown by the complete elimination of IL-8 production by basal THP-1 monocytes. * indicates P<0.05, when comparing individual treatments with control (no treatment) IL-8 production. ^#^ indicates P<0.05, when comparing individual treatments with 4hr. PPS + SBI IL-8 production.

C2BBe1 membranes were incubated with 0.01% PPS for 4 hours (without the addition of SBI) prior to beginning steric exclusion experiments to ensure that membranes were permeabilized prior to experimentation (70% TER reduction—Fig. 6), as previous experiments showed the concurrent addition of SBI with PPS inhibits PPS induced membrane damage (Fig. 4). PPS stimulated a significant increase in IL-8 production as the concentration of IL-8 was observed to increase from 280 ± 68 pg/mL in undamaged control cultures to 612 ± 55 pg/mL in cultures treated with 0.01% PPS alone. Similar to the results shown in Fig. 5, the addition of Pam3CSK4 to the apical compartment of PPS-damaged membranes resulted in antigen translocation, leading to an increase in IL-8 production from 612 ± 55 pg/mL (PPS only treatment) to 8545 ± 440 pg/mL (Fig. 6). Experimental variation in the absolute concentration of background IL-8 production by THP-1 monocytes was observed (Figs. 5–6). However, trends in IL-8 production for different treatment groups remained unchanged between repeated experiments. Pre-incubation of Pam3CSK4 with SBI for 1 hour prior to addition to the apical compartment led to a complete inhibition of IL-8 production by THP-1 monocytes (Fig. 6). The binding of Pam3CSK4 by immunoglobulins in SBI prevented Pam3CSK4 from translocating across the damaged epithelial barrier in this test system.

## Discussion

Serum-derived bovine immunoglobulin/protein isolate (SBI) is a unique plasma fraction that is enriched for immunoglobulins and intended for use in the management of chronic loose and frequent stools associated with GI enteropathies. Specifically, oral consumption of SBI is indicated for the dietary management of chronic loose and frequent stools associated with IBS-D and HIV enteropathy. The safety of this unique protein preparation has been evaluated in both pediatric and adult subjects [12,17,22,23], and SBI has been self-affirmed as Generally Recognized as Safe (GRAS) with no safety-related questions by the US Food and Drug Administration. We hypothesize that consumption of SBI floods the lumen of the GI tract with functional immunoglobulins capable of binding and neutralizing antigens involved with local immune activation. To test this hypothesis, immune exclusion and steric exclusion mechanisms were investigated in this study to better understand the benefits of oral immunoglobulin in reducing GI inflammation and maintaining gut homeostasis.

The immunoglobulin content of SBI has been increased from normal bovine plasma concentrations to greater than 50% IgG, 1% IgA, and 5% IgM. The high IgG content of SBI differs from the immunoglobulin pool at the mucosal surface of the GI tract which is comprised primarily of secretory IgA [24]. A primary benefit of secretory IgA in the digestive mucosa is its resistance to enzymatic digestion and retention of activity throughout the GI tract, whereas IgG is considered less resistant to enzymatic degradation. However, IgG has also been shown to be resistant to digestion and retain biological activity in the feces [25–28], suggesting that IgG is less susceptible to enzymatic digestion compared to other dietary proteins. Furthermore, previous work has shown that both IgG and IgA are capable of binding common antigens [29–32], which was similarly demonstrated in our study where three immunoglobulin isotypes were shown to bind LPS, Lipid A, and Pam3CSK4. Altogether, SBI is uniquely comprised of immunoglobulins which remain biologically active throughout the GI tract and have affinity for common intestinal antigens associated with GI inflammation.

Immunoglobulins are an essential part of the immune response and are typically generated against conserved regions or molecular patterns across microbial pathogens (e.g. LPS, flagellin, and viral structural proteins) [10,33,34]. Furthermore, bovine immunoglobulins have previously been shown to react with various antigens from bacterial and viral microorganisms known to cause serious illness in humans [10,15,35–39]. In this study the immunoglobulins found in SBI were shown to bind LPS, Lipid A, and Pam3CSK4 using a modified ELISA technique. LPS is highly prevalent among intestinal antigens due to the sheer magnitude of gram-negative bacteria comprising the microbiota. Lipid A is the hydrophobic antigenic head group of LPS which contributes to interactions with immune cells through toll-like receptor-4 (TLR4) to induce immune activation and cytokine production [40]. Pam3CSK4 is a synthetic TLR2 stimulator that mimics the N-terminal region of lipopeptides found in both gram-positive and gram-negative bacteria [41,42]. Binding of these antigens demonstrates the cross-reactivity of bovine immunoglobulins in SBI with several common antigens associated with GI tract inflammation in humans.

In addition to the immunoglobulins in SBI, albumin and transferrin are the next most abundant proteins. Albumin and transferrin are primarily involved with nutrient binding and transport [43–46], while albumin also has been shown to have some binding affinity for bacterial LPS [47]. Immunoglobulins are the largest functional protein component of SBI (~60% of the total protein) and are primarily responsible for antigen recognition and binding in the body. Therefore, we suspect that the most significant contributor to SBI binding of LPS, Lipid A, and Pam3CSK4 can be attributed to the immunoglobulins within SBI. Further evaluation of immune and steric exclusion and anti-inflammatory mechanisms with individual protein species will continue to be the focus of future research.

Immune exclusion and steric exclusion mechanisms have been hypothesized to result following immunoglobulin binding of antigen. The focus of this study was to understand how these mechanisms may be involved in reducing inflammatory cytokine production in response to antigen exposure. Immune exclusion occurs when immune cells are prevented from recognizing antigen bound with immunoglobulin. Immune exclusion of LPS and Lipid A through binding with the immunoglobulins in SBI was shown to be dependent on SBI concentration at constant antigen concentration, indicating that cytokine production can be completely inhibited with sufficient concentration of SBI to bind critical levels of free antigen. Conversely, Pam3CSK4 continued to stimulate IL-8 cytokine production by THP-1 monocytes when bound by immunoglobulins in SBI. These results indicate that immunoglobulin binding may modulate antigen interaction with cell surface receptors through multiple mechanisms. It is important to note that LPS interacts with TLR4 through the co-localization of several accessory molecules prior to the entire complex binding with TLR4 on the cell surface [40]. Conversely, Pam3CSK4 is a TLR1/2 agonist that interacts directly with cell surface receptors [48]. Therefore, the observed immune exclusion of LPS and Lipid A by SBI may be a result of immunoglobulins limiting accessory molecule interactions with the antigen. Further understanding the role of the immunoglobulins in preventing antigen interaction with immune cells will be the subject of future studies.

Steric exclusion is an additional mechanism that may further limit immune activation by controlling the movement of antigen across a damaged intestinal epithelial barrier. Steric exclusion refers to a change in the physical properties of an antigen (e.g. size, charge, etc.) such as might occur with immunoglobulin/antigen complex formation. In our study we demonstrated antigen translocation across a damaged epithelial barrier using a co-culture model that employed a C2BBe1 monolayer to separate an apical compartment from a basal compartment containing THP-1 monocytes. *In vitro* models, particularly Caco-2 monolayer cultures, have long been employed to study intestinal epithelial permeability and therapeutic absorption [49,50]. The THP-1 monocytes maintained in the basal compartment respond to each of the antigens and signify the presence of antigen by the production of measurable quantities of IL-8.

The permeability of a C2BBe1 monolayer can be altered through exposure to cytokines [51–54], the addition of EDTA and EGTA [55,56], or other chemicals [55]. In this study, the permeability enhancer palmitoyl dimethyl ammonio propanesulfonate (PPS) was used to damage C2BBe1 epithelial monolayers. Studies described by Gupta et al. indicate that PPS increases therapeutic macromolecule transport across the intestinal barrier by modulating tight junctions which increases the paracellular space between cells [19]. Damaging the C2BBe1 epithelial barrier with 0.01% PPS added to the apical compartment of the co-culture resulted in a 60–70% reduction in TER. Pam3CSK4 was the only antigen tested able to translocate the PPS damaged C2BBe1 barrier. The inability of LPS to translocate the damaged membrane may have been due to size, since the LPS used in these studies ranged from 10–20 kDa making it a fairly large molecule to move through damaged tight junctions. While both Lipid A and Pam3CSK4 are much smaller in size (1.5 kDa), the selective translocation of Pam3CSK4 and not Lipid A indicates that size is not the sole determining factor in antigen translocation across PPS-damaged membranes. A major difference between Lipid A and Pam3CSK4 is the inclusion of four terminal lysine residues on Pam3CSK4 giving this antigen a charged head group, whereas Lipid A is uncharged. Analysis of the permeability results indicates the selective permeability of PPS damaged barriers is a result of both antigen size and molecular charge.

Solute translocation across a damaged epithelial barrier is typically associated with either transport through the pore pathway or the leak pathway due to reorganization of the various major tight junction proteins belonging to the claudin and occludin families [57]. Opening the pore pathway leads to increased permeability of small charged solutes, whereas the leak pathway is less selective and allows larger macromolecules including LPS to translocate the membrane [4,57–59]. Based on antigen translocation results shown in Fig. 5, our data would suggest that PPS opens the pore pathway allowing small charged molecules to translocate an epithelial barrier while the leak pathway is likely unaffected. The defects in an epithelial barrier associated with the pore pathway have been estimated to have radii near 4Å, depending on cell type [57,60,61]. While the hydrodynamic radius of Pam3CSK4 is unknown, a PEG oligomer with similar molecular weight has a theoretical hydrodynamic radius of 8Å [60]. The hydrodynamic radius of Pam3CSK4 may be larger than that typically associated with the pore pathway; however Pam3CSK4 includes four lysine residues creating a charged head group. Poly-L-lysine peptides have been shown to confer increased cellular permeability and have been employed in many drug delivery applications [62]. Therefore, we hypothesize the charged poly-L-lysine head group may contribute to Pam3CSK4 translocation through the pore pathway opened by PPS treatment.

Pam3CSK4 was the only antigen tested that was able to translocate PPS-damaged C2BBe1 monolayers. Furthermore, Pam3CSK4 stimulation of THP-1 monocytes was unaffected by binding with immunoglobulins in SBI, an important characteristic providing that any Pam3CSK4 (either unbound or bound by the immunoglobulins in SBI) which translocates the C2BBe1 monolayer can be identified by monitoring THP-1 IL-8 production. Using these properties of Pam3CSK4 and the co-culture model it was possible to demonstrate the steric exclusion of Pam3CSK4 following binding with the immunoglobulins in SBI. The addition of an immunoglobulin/Pam3CSK4 complex to the apical compartment of the co-culture completely mitigates any antigen translocation across a damaged membrane. Steric exclusion occurs when the physical properties of an antigen (e.g. size, shape, charge) are altered through immunoglobulin binding. Assuming the typical IgG is around 150 kDa, the molecular weight (size) of the Pam3CSK4/immunoglobulin complex would be effectively increased nearly 100X following IgG binding. Additionally, the larger immunoglobulin could mask the charge on Pam3CSK4 if the poly-lysine charged head group lies within an active-site fold of the immunoglobulin. Changes in antigen physical properties could dramatically affect translocation across a damaged membrane. Although immunoglobulin binding with Pam3CSK4 does not directly prevent stimulation of THP-1 monocytes, inflammatory cytokine production is indirectly prevented by ensuring the antigen is incapable of binding with receptors on immune cells as it is sequestered to the apical compartment.

## Conclusion

The results presented here suggest that oral administration of immunoglobulins in SBI may reduce pro-inflammatory cytokine production by multiple mechanisms: 1) immune exclusion where immunoglobulin/antigen binding limits the ability of an antigen to interact with and activate immune cells, and 2) steric exclusion where immunoglobulin/antigen binding reduces antigen permeability across a PPS damaged membrane by altering the physical characteristics of an antigen. These results begin to explain how the immunoglobulin in SBI can positively affect GI inflammation and further our understanding of the complex interactions between oral immunoglobulins, antigens, and the immune system of the gut. Continued efforts to understand the downstream effects of immune and steric exclusion on GI disorders such as Crohn’s disease, ulcerative colitis and IBS will be the focus of ongoing research.

## Supporting Information

S1 Fig Binding Control Tests for IgA ELISA. (TIF)

S2 Fig Binding Control Tests for IgM ELISA. (TIF)

S3 Fig Specificity Control Tests for IgG ELISA. (TIF)

S4 Fig Specificity Control Tests for IgA ELISA. (TIF)

S5 Fig Specificity Control Tests for IgM ELISA. (TIF)
